# Allometry and Dissipation of Ecological Flow Networks

**DOI:** 10.1371/journal.pone.0072525

**Published:** 2013-09-03

**Authors:** Jiang Zhang, Lingfei Wu

**Affiliations:** 1 School of Systems Science, Beijing Normal University, Beijing, China; 2 Department of Media and Communication, City University of Hong Kong, Hong Kong, China; University Toulouse 1 Capitole, France

## Abstract

**Background:**

An ecological flow network is a weighted directed graph in which the nodes are species, the edges are “who eats whom” relationships and the weights are rates of energy or nutrient transferred between species. Allometric scaling is a ubiquitous feature for flow systems such as river basins, vascular networks and food webs.

**Methodology:**

The “ecological network analysis” can serve to reveal hidden allometries, the power law relationship between the throughflux and the indirect impact of node 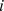, directly from the original flow networks without any need to cut edges in the network. The dissipation law, which is another significant scaling relationship between the energy dissipation (respiration) and the throughflow of any species, is also obtained from an analysis of the empirical flow networks. Interestingly, the exponents of the allometric law (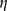) and the dissipation law (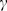) show a strong relationship for both empirical and simulated flow networks. The dissipation law exponent 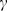, rather than the topology of the network, is the most important factors that affect the allometric exponent 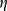.

**Conclusions:**

The exponent 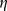 can be interpreted as the degree of centralization of the network, i.e., the concentration of impacts (direct and indirect influences on the entire network along all energy flow pathways) on hubs (the nodes with large throughflows). As a result, we find that as 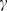 increases, the relative energy loss of large nodes increases, 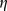 decreases, i.e., the relative importance of large species decreases. Moreover, the entire flow network is more decentralized. Therefore, network flow structure (allometry) and thermodynamic constraints (dissipation) are linked.

## Introduction

An ecosystem is an open thermodynamic system driven by energy flows [1]–[3]. Each unit of an ecosystem is a living organism and is also an open system in which free energy is embodied by nutrient flows in the blood that are distributed from the heart to the cells through the vascular networks [4], [5]. Moreover, all these units are connected by the so called food webs [6], on which energy flows are transported from environment to species and producers to consumers and are finally dissipated by respiration [2], [3].

Allometric scaling is one of the most important and significant empirical laws for such open systems driven by flows [7]–[10]. Kleiber discovered a fundamental allometry between metabolism and body mass [7]. Subsequent studies further extended the allometric scaling laws to the community and ecosystem level and found that many characteristics of species, such as population density [11], abundance and trophic levels [12] in the food web, can also be predicted by body size.

The origins of the allometric scaling law can be explained by the fractal branching transportation networks occurring throughout living systems [4], [5], [13]. The most efficient transportation networks embedded in a 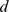-dimensional space, e.g., river basins in 2-d and vascular networks in 3-d, possess a ubiquitous power law, 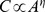, where 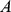 is the metabolism or input flows from the source to the network, 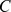 is the total “mass” or the summation of all individual flow rates in the network, and 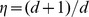 is the allometric exponent [5], [14], [15].

The food web, as the backbone of the ecosystem, can transport energy flow from the environment to each species [6], [16]–[18]. Do food webs show the allometric scaling pattern? Garlaschelli gave a positive answer by reducing empirical food webs to spanning trees on which 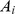 and 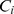 defined as the number of nodes and the summation of the 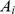
s on the sub-tree rooted from 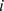, respectively, have a significant power law relationship with ubiquitous exponents of approximately 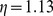 for almost all the food webs collected for the purpose of the analysis [19]–[21]. Thus, spanning trees from food webs have structures similar to the transportation networks in living organisms. One of the highly salient features of Garlaschelli's work is its focus on the network structure of food webs but not the individual or population of species as a whole as the metabolic theory usually does [9].

The energy flow information represented by the weights of the links in the network is, however, ignored by Garlaschelli's work, although this information is already available in some empirical data sets [22]. Actually, the studies of ecological flow networks concerning both “who eats whom” binary relationships and the “in what rate” problem [23] have produced remarkable findings [2], [24]–[26] and developed a sophisticated technique termed ecological network analysis [23], [27]–[30].

Both allometric scaling laws in metabolic theory and ecological network analysis can contribute to the understanding of energy flows. Nevertheless, they have hitherto remained separate. It appears that Garlaschelli's approach should fill the gap between the two theories. However, this approach does not help due to its own limitations: many edges must be cut to obtain a spanning tree, and the flow information on the edges is never considered [19], [31]. Therefore, it is crucial to extend Garlaschelli's method to generalized flow structures.

In this paper, we extend Garlaschelli's method by applying ecological network analysis to general flow networks through the calculation of the key variables 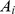 and 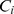 for each species in an ecological network. The allometric scaling relationships for 19 empirical ecological flow networks are presented in this study, and their allometric exponents are compared. In addition to the allometric scaling pattern, another interesting and significant empirical law called dissipation law is discussed. Dissipation, defined as the flux due to the respiration of the whole population, is found to be scaled with the total throughflow of each species on the ecological network. If we treat dissipation and total throughflow as the metabolic flux and body size of the whole population, the dissipation law can be viewed as an allometry at the population level.

In addition, a negative correlation between the allometric exponents (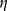
s) and dissipation law exponents (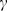
s) is uncovered for the empirical networks collected. A method to perturb the flow distributions on original ecological flow networks is then developed to further investigate the scaling relationship linking 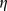 and 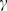. The inverse relationship of 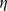 and 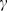 is further confirmed through a large number of experiments on both empirical and artificial ecological networks. We conclude that 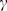 plays more important role than network topology in determining the allometric exponent.

This paper is organized as follows: Garlaschelli's approach and its extensions to general flow networks are reviewed in Section Methods. The so called Flow Adjusting Algorithm, the perturbation method applied to ecological flow networks to study the relationship between 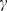 and 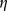, is introduced in Subsection Flow Adjusting Algorithm. The allometric scaling rules and the dissipation laws for 19 empirical ecological flow networks are shown in Section Results. Also, the scaling relation between 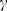 and 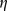 both on empirical and simulated flow networks is presented. Finally, we re-interpret the exponent in Section Discussion as the indicator of network centralization, i.e., the degree of the concentration of impacts on hubs in the network.

## Methods

### Review of Garlaschelli's Method

**Figure 1:**
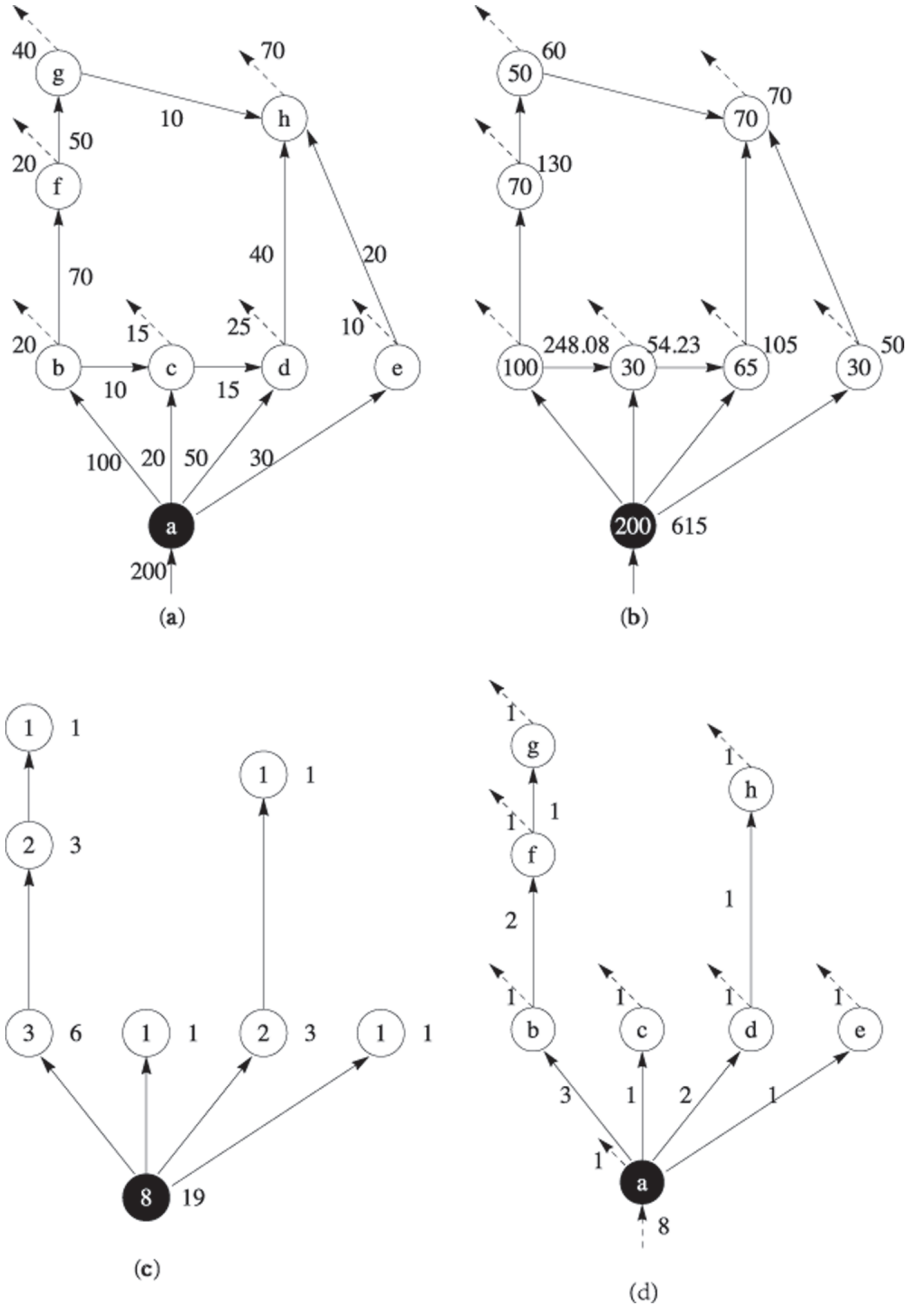
Comparison of different methods for calculating the allometric scaling of a hypothetical ecological flow network. (a) is a hypothetical network (the letter in each node is its index). The black node is the root, the numbers besides edges stand for flows, and the dashed lines represent dissipations; (b) shows the 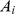, and 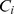 values inside and beside node 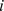, respectively, by our method; (c) is a spanning tree of (a), and the numbers inside and beside vertex 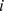 are the 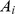 and 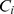 values calculated by Garlaschelli's method; (d) is the implicated flow network of (c), and the numbers beside the edges are the flux values.

To clarify the contribution of our method and its connection with current methods, we first review Garlaschelli's method for a hypothetic food web. Figure 1(a), (c) shows how Garlaschelli's approach can be applied to a hypothetical flow network (a). At first, a spanning tree (Figure 1 (c)) is constructed from the original network (Figure 1 (a)) by cutting edges. In this way, each sub-tree rooted from any 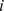 can be viewed as a sub-system of the spanning tree. 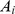 is the total number of nodes involved in this sub-tree and 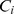 is the summation of the 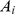
s for each node in this sub-tree. Finally, the universal allometric scaling relationship of the 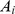
s and 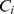
s, with an exponent approximately 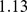, was found for all food webs considered [19].

Garlaschelli's method was inspired by the Banavar's model, which was developed to explain Kleiber's law [5]. The spanning tree is simply Banavar's optimal transportation network. Thus, energy flows into the entire system from the root along the links of the network to all the nodes. Suppose that each node consumes 1 unit of energy during each time step. A flux with 1 unit representing the energy consumption of each node should then be added to the original spanning tree (the dotted line in Figure 1(d)) [5]. As a result, the 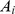 of each node is the throughflow of this node. 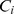 is the total throughflows of the sub-tree rooted from 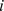. Essentially, the calculation of allometric scaling using Garlaschelli's approach is based on this weighted flow network model [31].

### Ecological Network Method

We will extend Garlaschelli's method directly on the original weighted network without cutting edges (e.g. Figure 1(a)). However, the key question is how 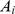 and 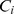 are to be calculated for a general flow network.

According to the flow network interpretation of Garlaschelli's method in Figure 1 (d), 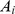 is the energy flux intake by node 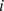, which is balanced with the total out flows through 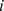. Thus, this concept can be extended to any flow network by defining 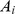 as the throughflow of node 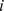 [27]. However, 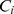 is not as straightforward to define as 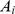 because the sub-system concept is not applicable if the considered network is not a tree.

To understand the meaning of 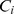 in Garlaschelli's method based on the flow network picture (Figure 1 (d)), we invoke the following hypothetical experiment. Assume that a large number of particles are flowing along the network (Figure 1 (d)), and that all particles passing any node, say b, will be attached a label, e.g., “b”. This trace marker will not be erased permanently unless the particle flows out of the network. We then find 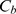 in Figure 1 (c) is simply the total number of particles labeled “b” that remain trapped in the entire network. This trace marker experiment can be also applied to other nodes independently and separately by attaching different labels so that all 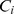
s can be calculated by counting the number of particles that ever passed node 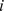.

This understanding can be extended to any flow network, whether or not it is a tree [31]. Although counting the number of labeled particles in the real network is difficult, we can perform this calculation directly with Markov chain techniques and the ecological network analysis developed by Patten et al [27], [28]. As long as the flow network is in a steady state, so that all flows distributed on edges are stable, a fixed 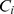 value can be calculated according to the flow structure.

Suppose that the flux matrix of the original network is 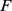, in which each entry 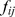 stands for the flux from 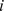 to 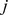. Two special nodes, 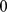 and 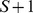 (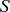 is the total number of species), representing the source and the sink, are contained in this matrix as the first(last) column(row). For most of the known ecological flow networks, the flows are balanced, i.e.,
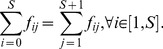
(1)


The webs not satisfying this condition will be balanced artificially by the method mentioned in Section A in File S1. Then, on a balanced flow network 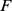, we define 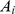 of 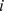 as
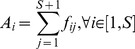
(2)


which is the throughflow of species 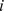 in ecological network analysis [32].

To calculate 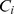, we should convert the original flux matrix 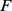 into a Markov chain 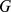 in which each element is defined as 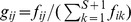 for all 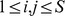. Thus, 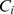 can be calculated as,
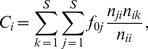
(3)where, 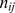 is an element of matrix 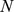, termed the fundamental matrix [23], [33] and defined as
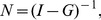
(4)where, 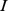 is the identity matrix. According to the ecological network analysis method, 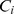 is simply the total number of particles that ever passed node 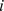 (see Section B in the Supporting Information).

We then calculate 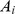 and 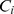 values for all nodes in the flow network to test the following allometric scaling law:
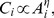
(5)where, 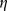 is the allometric exponent that will be primarily discussed in the following sections.

Another significant scaling relationship found in this paper is the dissipation law,
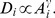
(6)


In this equation, 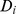 is the flux from species 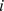 to the sink in the original flux matrix before the imposition of the artificial balance. This value can be read directly from the original data as follows:
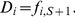
(7)


In ecological flow network, this flux represents the respiration and output of species 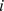. 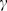 is the dissipation exponent, which must be estimated from the data.

### Flow Adjusting Algorithm

An important finding of this paper is the negative correlation between 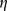 and 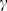. In addition to plotting the relationship between 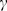 and 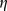 of the original flow networks directly, we also study the way in which the allometric scaling changes with the dissipation law by tuning the dissipation exponent without changing the topology on the networks. We devise a specific approach termed the Flow Adjusting Algorithm (FAA), to accomplish this goal.

Concretely, for a given flow network 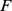, we keep the network topology and the ratio of influx (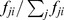) unchanged, but adjust the flow distributions on edges to obtain a new flow network 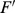 such that:

(1) The given dissipation law, i.e., 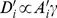, holds for every node 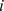 in 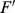, where 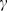 is a given exponent which can be tuned. We will study how 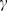 impacts the allometric scaling law.
(2) The flux balance condition, i.e., 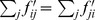, must be maintained for each node 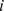.

The two requirements can be formalized by the following equations:
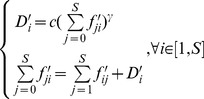
(8)where 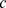 and 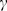 are given constants and the 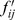
s and 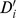
s are unknown variables. Therefore, we have a total of 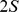 equations but 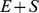 variables (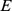 is the total number of edges). For most flow networks, 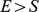, so infinite number of solutions to Equation 8 can be obtained. However, finding the solutions of these equations is still difficult due to their nonlinear nature.

The FAA is an approximate algorithm for solving these equations with the relative influx ratio of each edge unchanged. Suppose we have a node set 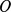. Initially, we set time step 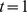, 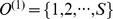 and the flux matrix as the original flow network 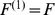, where the superscript on 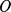 and 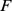 is the current time step. The algorithm will repeat the following steps:

(1). For any node 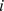 in 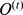, the algorithm needs the current out flows from 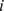, 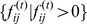. Solve the following equation for 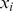:

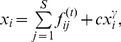
(9)i.e., the new total influx to 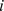. The solutions to these equations ensure that all nodes satisfy the flow balance condition and dissipation law.

(2). Assign 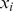 (the solution of Equation 9) to all incoming edges to 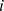 proportionately, set

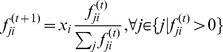
(10)and set,

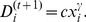
(11)
Equation 10 guarantees that the ratio of every influx to the total influx remains unchanged.

(3). Add all input nodes *j*s of 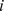 into set 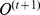 and delete 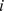 from 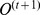.
(4). Set 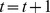 and repeat the previous steps until the stopping criteria, which include the requirements that the total running time is less than a given value and that the dissipation law exponent 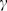 and 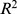 for the new flux matrix 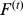 are close to the desired values, are satisfied.

This algorithm works only if the network is connected (i.e., there is at least one path from 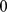 to every node 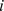). For most networks, the algorithm can converge to a network satisfying the significant dissipation law (the R square of the power law regression between 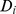 and 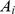 is greater than a given value) with the given 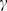 exponent. However, it may oscillate on some topologies, especially for random networks (see Section D in the Supporting Information).

## Results

### Description of Data Set

Nineteen ecological flow networks for different habitats are considered in this study(Table 1). The networks are obtained from the online database(http://vlado.fmf.uni-lj.si/pub/networks/data/bio/foodweb/foodweb.htm). Most of the networks in this database are from the literature [25], [34]–[39]. In Table 1, we list the name, the number of nodes (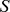) and the number of edges (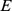) of these networks. Note that the nodes are either living species or non-living compartments, and the weighted links are energy flows whose values vary substantially because the units and time scales of the measurements are very different. The source node 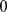 and the sink node 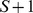 are the “input” node and the combination of “respiration” and “output” nodes in the original data, respectively. The dissipative flux of each node 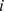 is simply the flow from 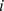 to 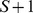 which can be read directly from the data. Most of the ecological flow networks considered are already balanced. The few unbalanced networks have been balanced with the approach cited in Section A of the Supporting Information.

**Table 1:** Empirical Ecological Flow Networks and Their Scaling Exponents.

Ecological Networks	Abbre.	*S*	*E*	*η*	R_η_^2^	*γ*	R*_γ_* ^2^
Florida Bay, Dry Season	Baydry	126	2102	1.010	0.995	0.915	0.949
Florida Bay, Wet Season	Baywet	126	2071	1.020	0.994	0.917	0.953
Mangrove Estuary,							
Dry Season	Mangdry	95	1462	1.010	0.997	0.978	0.983
Everglades Graminoids,							
Dry Season	Gramdry	67	863	1.030	0.999	0.973	0.997
Everglades Graminoids,							
Wet Season	Gramwet	67	863	1.020	0.999	0.977	0.998
Cypress,Dry Season	CypDry	69	639	0.998	0.996	0.957	0.949
Cypress,Wet Season	CypWet	69	630	0.997	0.997	0.965	0.988
Mondego Estuary							
-Zostrea site	Mondego	44	401	1.010	0.999	0.979	0.997
St. Marks River (Florida)	StMarks	52	349	1.020	0.980	0.985	0.950
Lake Michigan	Michigan	37	210	1.010	0.999	0.995	0.999
Narragansett Bay	Narragan	33	194	1.010	0.991	0.813	0.942
Upper Chesapeake							
Bay in Summer	ChesUp	35	203	1.050	0.997	0.952	0.991
Middle Chesapeake							
Bay in Summer	ChesMiddle	35	195	1.040	0.996	0.851	0.761
Chesapeake Bay							
Mesohaline Net	Chesapeake	37	160	0.994	0.997	0.985	0.985
Lower Chesapeake							
Bay in Summer	ChesLower	35	163	1.050	0.997	0.926	0.971
Crystal River Creek							
(Control)	CrystalC	22	107	1.040	0.997	0.959	0.995
Crystal River Creek							
(Delta Temp)	CrystalD	22	83	1.040	0.998	0.963	0.996
Charca de							
Maspalomas	Maspalomas	22	82	0.956	0.966	1.150	0.737
Rhode River Watershed							
- Water Budget	Rhode	18	54	0.828	0.866	1.200	0.963

### The Allometric Scaling Law

**Figure 2:**
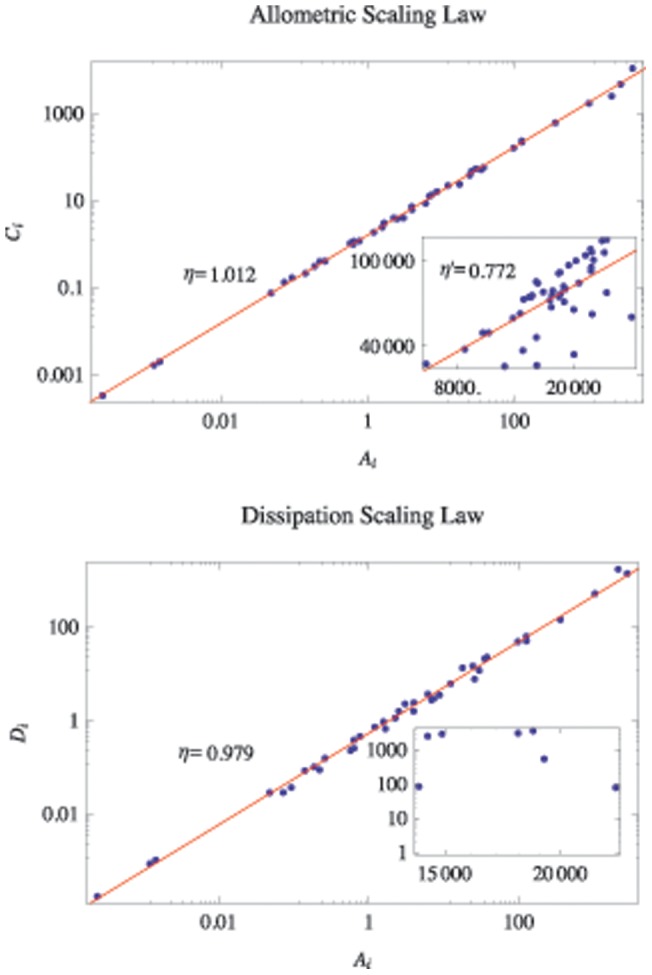
Allometric scaling law and dissipation law for the Mondego ecological network and the null model (insets). . Only a few points appear in the inset of the lower figure because many nodes in the null model are not balanced and their dissipation values are set to zeros.

We find that all the ecological flow networks possess a significant allometric scaling pattern. Their allometric exponents 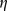 and the values of 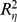 are listed in Table 1. The values of 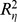 are generally greater than 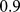 with the exception of the Rhode network, whose scale is relatively small (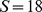). All exponents 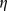 fall within the interval 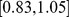. Most of these exponents are slightly greater than 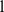. An example of the allometric scaling fitting is shown in Figure 2.

To test whether the allometric scaling pattern is significant compared to random flow networks, we build a null model in which the numbers of nodes and edges are maintained, all links are re-connected randomly and all flows on edges are also randomly assigned on the interval 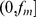 evenly, where 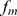 is the maximum flux of the original network. From the inset of the upper plot in Figure 2, we see that the null model network does not show a significant allometric scaling law. We also attempt to compare the empirical flow networks against other null models. Some of these null models are obtained by maintaining the topology unchanged but randomly assigning the weights, whereas others are obtained by simply shuffling the weights among the edges. All the details of these null models are presented in Section C of File S1.

Among these null models, the models in which the weight information is retained generally have allometric scaling exponents similar to the original values. As a result, we know that it is the flow distribution, but not the topological structure of the network, that plays the more important role. However, it is impossible to study all possible flow distributions that can affect the final allometric law because hundreds of flows can be adjusted for an empirical flow network. Fortunately, we find that the dissipation pattern of flows provides a key clue for understanding the allometric scaling.

### The Dissipation Law

In ecology, the dissipative processes of a species have different forms, such as respiration, excretion, egestion, and natural and predatory mortality [3]. In our data, the dissipative flow is primarily respiration. It is plausible that this output flow would increase with the total throughflow of the focal species. However, it is not obvious for most collected ecological flow networks that the growth of dissipation for different species is slower than the growth of throughflow. A sub-linear relationship (Equation 6) between the dissipation and throughflow of each species holds per se. The values of the 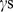 for different networks are also listed in Table 1. Note that almost all the 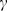 values are less than 1 with the exception of the Maspalomas and Rhode networks. The lower plot in Figure 2 shows the dissipation scaling law for the Mondego flow network as an example.

### The Relationship between 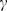 and 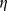


**Figure 3:**
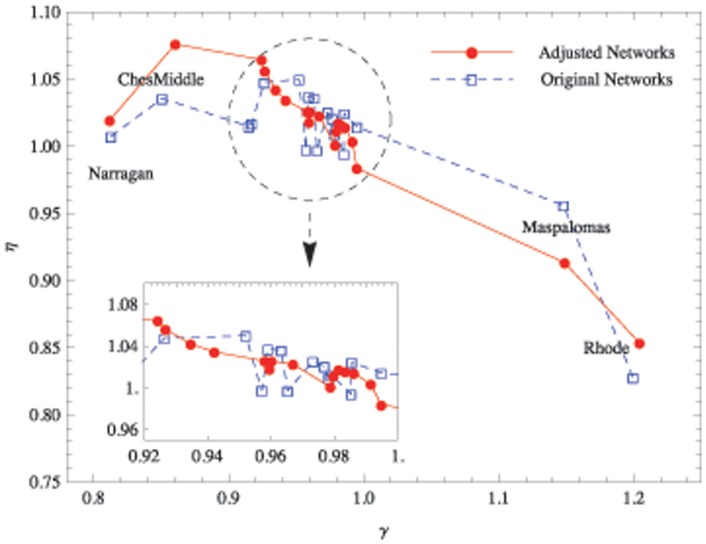
The relationship between 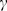 and 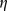 in the original and adjusted ecological flow networks. The blue dashed line is the 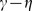 curve for the original flow networks, and the solid red line is the corresponding curve for the adjusted networks based on the same network structure and dissipation law according to the flow adjusting algorithm (see the main text).

Because both 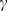 and 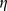 are indicators for the entire flow network, to determine how these two numbers are correlated with each other we can simply plot different 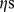 against 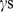 across all the collected empirical flow networks (see the blue dotted line in Figure 3). Although a general tendency for 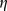 to decrease with 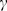 is observable, this trend is not significant for three major reasons. First, the number of sample points is too small to show a clear relationship. Second, most of the data points are concentrated in the circled area because all 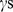 and 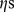 are similar in value for all networks. Finally, the original exponents are noisy, so that the 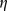 values fluctuate within the circled area.

**Figure 4:**
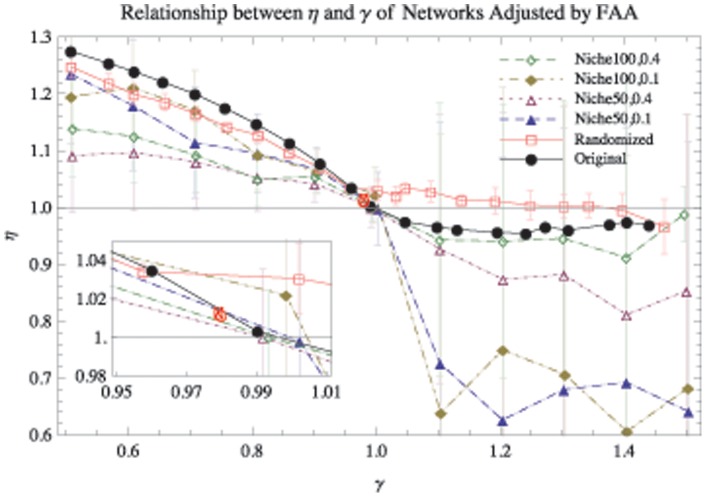
The relationship between 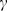 and 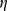 according to the flow adjusting algorithm (FAA) applied to the Mondego ecological network, the randomized Mondego flow network, and the networks generated by the Niche Model. The black filled circles and red hollow squares are the original and randomized Mondego flow networks, respectively. The randomized flow network retains the topological structure of the Mondego network but assigns the flux randomly. The other dashed curves are for the networks generated by the Niche model [40] with different numbers of nodes (100 or 50) and connectances (0.4 or 0.1), as well as for the randomly assigned flows. The FAA is applied to all these flow networks to obtain the relationships between 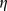 and 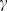. All the exponents represent the averages of the results of 50 random experiments. The red star(“*”) shows the original positions of the dissipation law exponent (0.980) and the allometric exponent (1.012) of the Mondego network. The red open circle corresponds to the combination of the original dissipation law exponent (0.980) and the adjusted allometric exponent (1.011) according to the FAA for the Mondego network.

To avoid the aforementioned problems, we investigate the relationship between 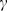 and 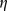 by perturbing the original networks using the FAA cited in Subsection Flow Adjusting Algorithm. In this way, we can perturb the flow structure of the original network to obtain the expected dissipation law and to observe how exponent 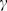 affects exponent 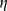. Figure 4 shows the dependence of 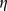 and 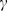 on the perturbed networks according to the FAA based on the Mondego flow network, a randomized Mondego flow network (based on the Mondego's topology but assigning flows randomly) and networks generated by the Niche model [40]. Note that the allometric scaling exponent decreases with the dissipation law exponent in a similar manner, whatever the original network structures. However, the shapes of the curves portraying the relationship between 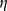 and 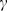 change with the network structures. For networks generated by the Niche model, 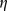 decreases less rapidly with 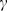 if the connectances are higher (blue filled triangles versus purple hollow triangles and yellow filled diamonds vs. green hollow diamonds). As a result, the dissipation law exponent, but not the structure, is the major feature affecting the allometric exponent. However, we cannot conclude that the topological structure has no influence on the allometric exponents. Section D of File S1 will discuss the relationships among structure, the dissipation exponent and the allometric exponent in details for spanning trees.

In Figure 4, the red hollow circle in the middle of the black filled curve shows the combination of the dissipation law exponent 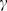 of the empirical Mondego network 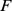 and the allometric exponent 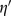 of the adjusted flux matrix 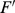 resulting from the FAA. The red star corresponds to the original exponents of the Mondego network for both 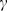 and 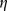. We see that these two markers almost overlap. This result means that the original Mondego food web satisfies the following two requirements:

(1) the dissipation law is significant for the original 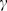;
(2) the balance equation, Equation 1, is obeyed exactly.

However, for other empirical networks, the perturbed result for the allometric exponent is not identical to the given original dissipation law exponent either because the flux balance requirement is violated or the dissipation scaling law is not significant (see Section E of File S1).

By adjusting the flows on all empirical flow networks with the fixed original dissipation law exponent 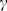, we can eliminate noise in the raw data. Because we require the networks to be flow balanced and satisfy the given dissipation law. The red solid curve in Figure 3 shows a clear relationship between 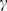 and 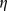 for all empirical flow networks. Note that the decreasing trend of the empirical exponents (blue dash line) is not as strong as that of the exponents obtained from FAA (red line). Therefore, the two requirements stated above are not satisfied perfectly by many empirical ecological networks.

## Discussion

### Transportation Efficiency or Degree of Centralization?

Previous studies have explained the allometric scaling exponent 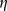 as the transportation efficiency of the network, with values ranging from 1 (a star network, the most efficient tree) to 2 (a chain, the most inefficient tree). However, in our study, the allometric scaling exponent is not restricted to the interval 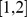. Therefore, we should offer a new explanation for the exponent 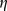. The key problem is to understand the indicator 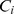.

In Banavar′s model and Garlaschelli's method, 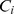 is characterized as a cost of energy transportation for the sub-tree rooted from 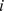 [5]. From this perspective, the energy flows on the redundant links (loops or cross-leveled links), except for the links in the spanning tree, are wasted. Nevertheless, this interpretation cannot be plausibly generalize to flow networks because (1) the wasted energy in the weighted networks can be measured as the dissipation of each node but not the weight of the edges and (2) all energy links should be considered because they all contribute to the overall flow distribution.

According to the particle tagging experiment described in Section Methods, we can interpret 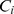 as the total impact of 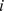 to the whole network along all flow pathways [31], [41] because it is the total number of particles passing 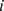 at least once. Thus, as 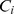 increases, additional nodes will be affected by the particles ever passed 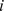, and the direct and indirect influences of 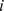 will increase. This interpretation of 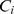 can be extended to any flow network.

As a species ascends up along the energy throughflow gradient 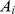, its total impact 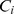 also increases with the relative speed 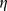 according to the allometric scaling law 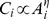. Therefore, the important nodes (with larger 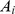) may have much greater power (total impact 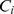) in the networks with larger exponents 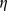 than in those networks with smaller exponents. For example, consider two networks with four nodes. They have the same throughflow distributions, e.g., 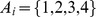, but different exponents 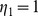 and 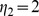. As a result, they have different 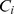 distributions, 
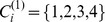
 and 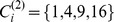. The most important node (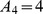) in the second network has a much greater impact (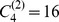) on the entire network than is the case in the first network (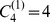). Hence, the concentration of the species impact of the second network is larger than that of the first network. Thus, the second network is more centralized than the first.

In short, the allometric exponent 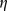 measures the degree of centralization of the network. The flow networks with exponent 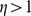 are centralized, whereas those with exponent 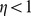 are decentralized.

This new interpretation is compatible with the previous one. For Garlaschlli′s spanning trees, the most centralized tree with a given root is a chain but the most decentralized structure is a star (see the discussion on spanning trees in Section D of the Supporting Information).

### Dissipation and Centralization

According to the new interpretation of the allometric exponent and the discussion of the relationship between dissipation and allometry, we can obtain an overall perspective: the networks may become more decentralized by dissipating more energy on larger nodes because the impacts of high flux nodes are weakened. In the networks with 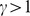, the dissipation flow per unit of throughflow increases with the throughflow, therefore the energy invested in the entire network decreases with the size of the node and the network is more decentralized. In contrast, if 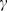 is less than 1, the dissipating flux scales to the throughflow with a smaller relative speed. So that the large nodes can output more energy to the entire network to obtain a much more powerful impact on other nodes, the networks are more centralized.

However, an interesting and still unexplained finding is that the allometric exponents of empirical ecological networks are all close to 1. They are neither centralized nor decentralized. We conjecture that this finding can be explained by an optimization between flow structure stability and energy transport efficiency. This hypothesis will require additional study.

## Concluding Remarks

In summary, this paper generalizes the universal allometric law to ecological flow networks and shows that the major factor influencing the allometric exponent is 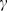, the dissipation law exponent. By reinterpreting the allometric exponent as the degree of centralization, we establish a connection between network structure and thermodynamic constraints. This connection is of substantial importance and deserves additional attention.

## Supporting Information

File S1
**Contains.** Figure S1. The relationships between 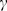 and 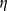 for different balancing methods. Naive, input, output, and average stand for different balancing methods. Figure S2. An example network showing the calculations of 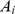 and 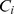. Figure S3. Allometric Scaling Patterns for Null Models based on the Mondego Network. Figure S4. Comparison of Allometric Scaling Exponents of Original Networks with the Null Models Figure S5. Dissipation Law for Null Models of Mondego Network. Figure S6. 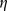 and 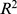 Change with 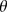, 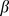 and 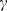. Figure S7. 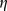 and 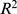 Change with 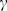 for different random networks. Figure S8. 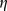 and 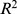 Change with 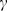 and 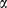 on the Random Networks based on Spanning Trees. Figure S9. 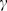 and 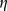 Relation Adjusted by the Flow Adjusting Algorithm on the Collected Ecological Networks. Figure S10. Dissipation Scaling Law for the Original and Balanced Flow Networks of Baydry and Rhode. Table S1. Unbalanced Flux for Each Ecological Network. Table S2. Comparison among Different Balancing Methods for Allometric Scaling Exponent.(PDF)
