# Supplementary material for: Allometry and Dissipation of Ecological Flow Networks
Source: PLoS One. 2013 Sep 3;8(9):e72525. doi: 10.1371/journal.pone.0072525 (PMC3760856; doi:10.1371/journal.pone.0072525)
Supplement: File S1 — Contains. Figure S1. The relationships between and for different balancing methods. Naive, input, output, and average stand for different balancing methods. Figure S2. An example network showing the calculations of and . Figure S3. Allometric Scaling Patterns for Null Models based on the Mondego Network. Figure S4. Comparison of Allometric Scaling Exponents of Original Networks with the Null Models Figure S5. Dissipation Law for Null Models of Mondego Network. Figure S6. and Change with , and . Figure S7. and Change with for different random networks. Figure S8. and Change with and on the Random Networks based on Spanning Trees. Figure S9. and Relation Adjusted by the Flow Adjusting Algorithm on the Collected Ecological Networks. Figure S10. Dissipation Scaling Law for the Original and Balanced Flow Networks of Baydry and Rhode. Table S1. Unbalanced Flux for Each Ecological Network. Table S2. Comparison among Different Balancing Methods for Allometric Scaling Exponent. (PDF) [file pone.0072525.s001.pdf]

Supporting Information of Allometry and Dissipation of Ecological Flow Networks

Jiang Zhang[1], Lingfei Wu[2,*]
1 School of Systems Science, Beijing Normal University, Beijing, China
2 Department of Media and Communication, City University of Hong Kong, Hong Kong, China
$*$ **E-mail: zhangjiang@bnu.edu.cn**

A Balancing A Flow Network

For most empirical ecological flow networks, the flux matrix F is balanced which means $\sum_{j=0}^{S} f_{ji} = \sum_{j=1}^{S+1} f_{ij}$ holds for each $i \in [1, S]$ (See Table S1 for the relative errors of unbalanced flows of each ecological network). However, some empirical networks and most artificial networks (e.g. random network) are always unbalanced. Therefore, we should balance the given network F artificially so that the ecological network analysis methods can be applied.

[Table 1 about here.]

Although some sophisticated balancing method had been developed [1, 2], we adopt a very simple approach which is called the naive method. Suppose $\sum_{j=0}^{S} f_{ji} \neq \sum_{j=1}^{S+1} f_{ij}$ for node i. We add an edge with the flux $|w|$, $w = \sum_{j=0}^{S} f_{ji} - \sum_{j=1}^{S+1} f_{ij}$ to connect node i to the sink (node $S+1$) or the source (node 0). If $w > 0$, the direction of this newly added edge is from i to $S+1$. If $w < 0$, the direction is from 0 to i. We can do this process for all nodes except 0 and $S+1$ to balance the entire network.

We also tried other methods mentioned in [2] and found similar results (i.e., similar allometric scaling exponent and the dependence between γ and η). The comparisons of the results by different balancing methods can be referred to Table S2 and Figure S1. Therefore, our results obtained in this paper are insensitive to the balancing methods. However, when we study the random networks and other null models, we hope that all flows are generated by the algorithms but not affected by the balancing method. As a result, the naive balancing method is the best choice because it never modifies the original flows.

[Table 2 about here.]

[Figure 1 about here.]

B Explanation on C_i Calculation by an Example Network

In this Section, we will explain why the number of particles labeled by node i can be calculated using Equation 3 in the main text with an example network (see Figure S2).

[Figure 2 about here.]

Notice that the original network is unbalanced, we should balance it at first by the approach mentioned in the last section. The balanced network then can be converted to a Markov chain G shown in Figure S2 (c). We know an element g_{ij} in Markov matrix G stands for the transfer probability of a particle from i to j, if the particle is located at i already and the entire network is in the steady state. Consequently, an element in matrix G^t stands for the probability of a particle transferring from i to j along all possible pathes of length t. All of the information is aggregated in the fundamental matrix N because,

$$N = I + G + G^2 + \cdots + G^\infty = (I - G)^{-1}. \quad (1)$$

We should be careful to give an intuitive explanation on N because its element n_{ij} is not a probability anymore, although the element in each term G^t is the transitional probability. We can write down the fundamental matrix for our example network (by ignoring the source and the sink),

$$N = I + G + G^2 + \cdots = \begin{pmatrix} 1 & 3/5 & 7/20 & 1/5 & 2/5 \\ 0 & 42/41 & 7/82 & 14/41 & 28/41 \\ 0 & 12/41 & 42/41 & 4/41 & 8/41 \\ 0 & 3/164 & 21/328 & 165/164 & 21/41 \\ 0 & 3/82 & 21/164 & 1/82 & 42/41 \end{pmatrix} \tag{2}$$

Notice that the elements on the diagonal are larger than 1, so they cannot be interpreted as probability.

Next, we will calculate the first passage flow P_i to any node i. Here, P_i is defined as the number of particles passing i the first time. This quantity can be calculated as,

$$P_i = \sum_{j=1}^{S} \frac{f_{0j} n_{ji}}{n_{ii}}. \tag{3}$$

For example, the first passage flow of node $4(P_4)$ can be calculated as,

$$P_4 = \sum_{j=1}^{S} \frac{f_{0j} n_{j4}}{n_{4,4}} = f_{0,1} \frac{n_{1,4}}{n_{4,4}} = 100 \times \frac{1/5}{165/164} = 656/33. \tag{4}$$

Actually, each term of Equation 3 is the first passage flow from node j to i, that is the number of particles that have visited j (in whatever time) and finally arrive at i in the first time. By dividing by the term n_{ii}, one avoids repeatedly counting the particles that have visited i [3].

Because at each time step there are totally P_i unlabeled particles passing i and be labeled by "b", thereafter, P_i new "b" particles will be injected to the system and will flow to other nodes along pathways. Then, at a given time step, there are totally P_i new labeled particles visiting i, $\sum_{k=1}^{S} P_i G_{\{ik\}}$ labeled particles visited i one time step ago, and $\sum_{k=1}^{S} P_i G^2_{\{ik\}}$ labeled particles visited two time steps ago,...,$\sum_{k=1}^{S} P_i G^t_{\{ik\}}$ labeled particles arriving at node i t time steps ago, and so forth.

Hence, the total number of labeled particles that flowing in the entire network at each time, being defined as C_i, is simply the summation of the labeled particles being attached 1 time step ago, 2 time steps ago,..., and so on. Therefore, we can calculate C_i as:

$$\begin{aligned} C_i &= \sum_{k=1}^{S} P_i (I + G + G^2 + \cdots + G^{\infty})_{\{ik\}} \\ &= \sum_{k=1}^{S} P_i N_{\{ik\}} \\ &= \sum_{k=1}^{S} (\sum_{j=1}^{S} f_{0j} n_{ji} / n_{ii}) n_{ik} \end{aligned} \tag{5}$$

For example, C_2 of node 2 in the example network is calculated as:

$$C_2 = P_2 \sum_{k=1}^{5} n_{2k} = ((100 \times 3/5 + 0)/(42/41)) \sum_{k=1}^{5} n_{2k} = 125 \tag{6}$$

C Null Models

To test if the allometric scaling law is a significant in empirical ecological network, we designed four kinds of null models based on empirical networks.

Null Model 1 (NM1): We keep the total number of nodes and edges the same as the original empirical flow network, set up random connections, and assign random weights for each edge. The weights are evenly distributed on the interval $(0, f_m]$, where f_m is the maximum flux in the original network. In this way, both of the topology and the flow distribution are changed.

Null Model 2 (NM2): The connections are kept, but the weights are randomly assigned for each edge. In this model, weights are also randomly sampled from the interval $(0, f_m]$. In this way, only the flow distribution is changed.

Null Model 3 (NM3): Keep the connections and shuffle the weights on edges. That is, we keep the topology and weights distribution but permute these weights on edges. In this way, the flow distribution is not changed but the correlations between flows are changed.

Null Model 4 (NM4): Keep the weights, the number of edges, but randomly assign the weighted connections between each pair of nodes. In this way, the flow distribution is kept, but their correlations and the network topology are changed.

For each original ecological flow network, we build four null models. The flux matrix F of the null models is unbalanced normally, then we should balance it by the approach mentioned in Section A. After that, we calculate A_i and C_i for each node and derive the allometric scaling law pattern.

[Figure 3 about here.]

Figure S3 shows the allometric scaling relationships between A_is and C_is for null models of the Mondego network. From this figure, we know NM3 and NM4 have more similar patterns as the original networks than NM1 and NM2. That means the flow distribution is more important than the topology in determining the allometric scaling. Although NM3 and NM4 have significant scaling pattern, their exponents ηs are smaller than the one of the original Mondego network. Therefore, NM3 and NM4 cannot reproduce the main characters of the original network.

Furthermore, we generate 50 networks for each null model on each collected empirical flow network. The average values of ηs are compared to the original ones in Figure S4. From this figure, we can see that the exponents of NM3 and NM4 are more close to the original networks with less fluctuations. That means the weights information is more important than the structures and the weights correlation play a minor role on allometric exponents.

[Figure 4 about here.]

[Figure 5 about here.]

Further studies on the dissipation laws of these null models can explain the patterns shown in Figure S4. Figure S5 shows that most networks do not show obvious dissipation scaling law. However, a straight lines can be observed from the ceilings of data clouds for NM3 and NM4. Actually, it is the result of the artificial balancing method can explain this phenomenon. Because all weights of NM3 and NM4 are not changed, but the connections are altered so that the energy influx cannot balance with the out flows for many nodes. As a result, the balanced flows (dissipations) are almost proportional to the original flows. Before we fit the data clouds by using a line on NM3 and NM4, we actually treat the data points under the lines as outliers. In this way, we can estimate the right γs for NM3 and NM4 which are close to 1. By comparing Figure S5 and Figure S3, we know that the negative correlation between γ and η is also suitable for these null models.

D γ and η Relationships for Modeled Networks

To better understand how γ correlates with η, we study several special modeled networks in this section.

D.1 Minimum Spanning Tree

We now consider the minimum spanning trees introduced in [4]. By controlling two parameters θ and β, we can generate variant trees with different basal species ratio(controlled by β) and maximum trophic level (controlled by θ).

The tree's construction process is as follows. Let's consider an ecological community with S different species, in which a hypothetic food web (tree structure) will be built. At first, we select βS species as the basal species at the first trophic level. At each time, a new species j is added to the minimum spaning tree. j will select a node i as its unique prey with the probability:

$$\Pi_{ij} = \frac{t_j^{-\theta}}{\sum_{k \in T} t_k^{-\theta}}, \tag{7}$$

where, t_j is j's trophic level + 1 (i.e., the depth of j from the root in the tree), T is the set of species which are already in the spanning tree, and θ is a parameter to control the attachment preference of the new node on depth. If θ is large, the new node may attache to the position closed to the root.

After a tree is constructed, we will assign random weights in the original flux matrix and then apply the FAA on it. In this way, we can investigate the influence of both tree's structure (θ and β) and dissipation exponent γ on the allometric scaling exponent η.

[Figure 6 about here.]

From Figure S6, we found at first both the dissipation law and network structure can affect the allometric scaling exponent. However, η may change more sharply when γ changes, compared to the changes of β and θ. When γ is given, the similar dependence η on β and θ as introduced by [4] can be observed.

Interestingly, when γ is set to 0, each node's dissipation is a constant, this corresponds to Garalaschelli's approach's assumption (see Figure 1(d) in the main text). And the exponent η derived by our algorithm is identical to the result derived by Garlaschelli's approach on the same tree. And the dependence of η on β and θ is also same as the results in [4]. Consequently, our method can recover Garlaschelli's method on spanning trees.

D.2 Random Network

We also test the "Flow Adjusting Algorithm" on random networks. The results are shown in Figure S7.

[Figure 7 about here.]

Only the results of random networks with 50 nodes containing different number of edges are shown, because the FAA is difficult to converge on the random networks with a large number of edges. When the algorithm cannot get a final result after a given number of time steps (200), we have to regenerate a new random network with the same number of nodes and edges. It is interesting that when the number of edges is large, the responding curve of η on γ is very different from the ones in Figure 4 of the main text. ηs are always small, and there is a peak when γ approaches 1. Therefore, we know the topological structure does affect the allometric exponent.

Furthermore, we generate random networks based on a minimum spanning tree by adding $\alpha(S(S-1)/2 - S)$ additional edges randomly. When $\alpha = 0$, the network is a minimum spanning tree, however, when $\alpha = 1$, it is a complete graph. Therefore, we can observe how the dependence of η on γ changes when the network structure changes from a tree to a random network by tuning α.

[Figure 8 about here.]

From Figure S8, the dependance of η on γ when $\alpha = 0.4$ is different from the random networks with the same connectance. But, it is similar to the ones of the empirical ecological networks. That is because the former random network is generated based on a minimum spanning tree which can be viewed as its backbone. The backbone tree's structure can take effect on the allometric exponent.

According to these experiments, we know both network structure and dissipation law exponent can influence allometric exponents. The exponent γ is more important than the structure. And the shape of backbone spanning trees can change the dependence between η and γ.

E Flow Adjustment for Empirical Ecological Flow Networks

The FAA has been applied to collected empirical ecological flow networks. The result of Figure 4 in the main text is for the Mondego network as an example. In this section, we will show the results for other networks and discuss the technique details.

[Figure 9 about here.]

The basic idea of the FAA is to tune various flows on the network until the dissipation law exponent is close to the desired value. When we apply this method to the empirical ecological networks, we adjust the flows until either ((1) the adjusted dissipation law exponent γ' satisfies $|\gamma' - \gamma^*| < 0.01$ (where γ^* is the desired exponent) and (2) the R'^2 of the dissipation power law on the adjusted flows should satisfy $R'^2 \leq R_\gamma^2$ (where R_γ^2 is the Rsquare of the dissipation law for the original flow network)); or (3) The running time steps are greater than 500. Because the algorithm may diverge whence the first requirement may not be satisfied, we have to stop the algorithm within a finite time steps and retrieve one of the best network as the output.

In Figure S9, we show results of applying the FAA on all collected ecological networks. The red diamond and green stars are the γ and η combinations for the original networks, and the red disk and green squares stand for the ones of the FAA results on the original γ as the designed exponent. If the original flow network satisfies the dissipation law and the flow balance condition perfectly, then the adjusted η value should be similar with the value of the original network (which means that the red disks(green squares) should overlap with the red diamonds(green stars)). However, we observe that the markers for some networks do not overlap. That means the corresponding original networks do not satisfy that two conditions perfectly.

[Figure 10 about here.]

Let's take the Baydry network as an example. Although the original flow network possess a very good dissipation law as shown in the left-top plot in Figure S10, this network is not balanced. Because the balance condition is a basic requirement of the allometric scaling law calculation, we have to balance the Baydry network at first by the method in Section A. However, the balanced flow network always has different dissipation law as the original one (compare the left-top plot to the right-top one in Figure S10). Therefore, the original η and the one adjusted by the FAA are different (The red disk and diamond in the first plot of Figure S9).

Another example network which is balanced, but not follow the good dissipation law, is Rhode as shown in the left-bottom and right-bottom plots of Figure S10. It is shown that the dissipation scaling law is not significant ($R^2 = 0.737$) for the Rhode network. Therefore, the original and adjusted exponents do not overlap (The first plot green star and square in Figure S9). Additionally, the FAA cannot generate a convergent result on the Rhode network. That is the reason why the green star and square have different horizontal coordinates.

Because the negative relationship between γ and η is significant only if the flow network satisfies (1) a significant dissipation law and (2) the flows are balanced, we adjust the flows of the empirical networks to obey the dissipation law with the original dissipation law exponent. In this way, we believe the noise contained in original data can be eliminated, so that more exact allometric exponents can be computed. The red solid curve in Figure 4 of the main text shows the original γ exponents and adjusted η exponents.

References

1. Savenkoff C, Vezina AF, Bundy A (2001) Inverse analysis of the structure and dynamics of the whole newfoundlandclabrador shelf ecosystem. Technical report, Canadian Technical Report of Fisheries and Aquatic Sciences 2354.

2. Allesina S, Bondavalli C (2003) Steady state of ecosystem ?ow networks: a comparison between balancing procedures. Ecological Modelling 165: 221-229.

3. Higashi M, Patten BC, Burns TP (1993) Network trophic dynamics: the modes of energy utilization in ecosystems. Ecol Model 66: 1–42.

4. Frank F, Murrell D (2005) A simple explanation for universal scaling relations in food webs. Ecology 86: 3258–3263.

List of Figures

S1 The relationships between γ and η for different balancing methods. Naive, input, output, and average stand for different balancing methods [2] 8
S2 An example network showing the calculations of A_i and C_i 9
S3 Allometric Scaling Patterns for Null Models based on the Mondego Network 10
S4 Comparison of Allometric Scaling Exponents of Original Networks with the Null Models . 11
S5 Dissipation Law for Null Models of Mondego Network 12
S6 η and R^2 Change with θ,β and γ 13
S7 η and R^2 Change with γ for different random networks 14
S8 η and R^2 Change with γ and α on the Random Networks based on Spanning Trees 15
S9 γ and η Relation Adjusted by the Flow Adjusting Algorithm on the Collected Ecological Networks 16
S10 Dissipation Scaling Law for the Original and Balanced Flow Networks of Baydry and Rhode 17

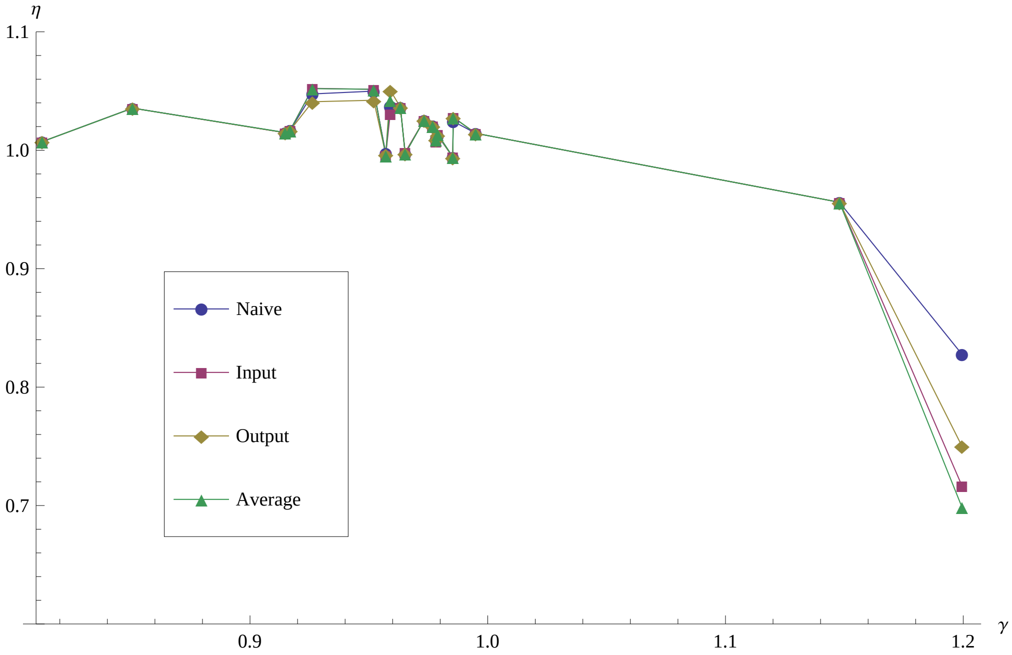

Figure S1. The relationships between γ and η for different balancing methods. Naive, input, output, and average stand for different balancing methods [2]

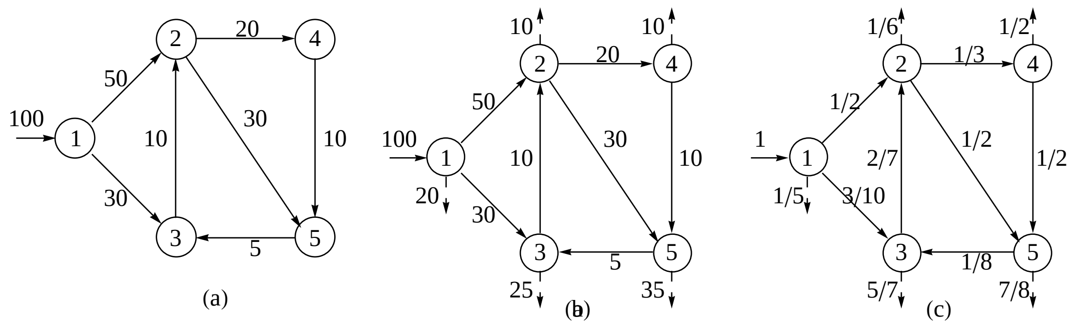

Figure S2. An example network showing the calculations of A_i and C_i
(a). The original flow network which is unbalanced; (b). The balanced network; (c). The corresponding Markov chain

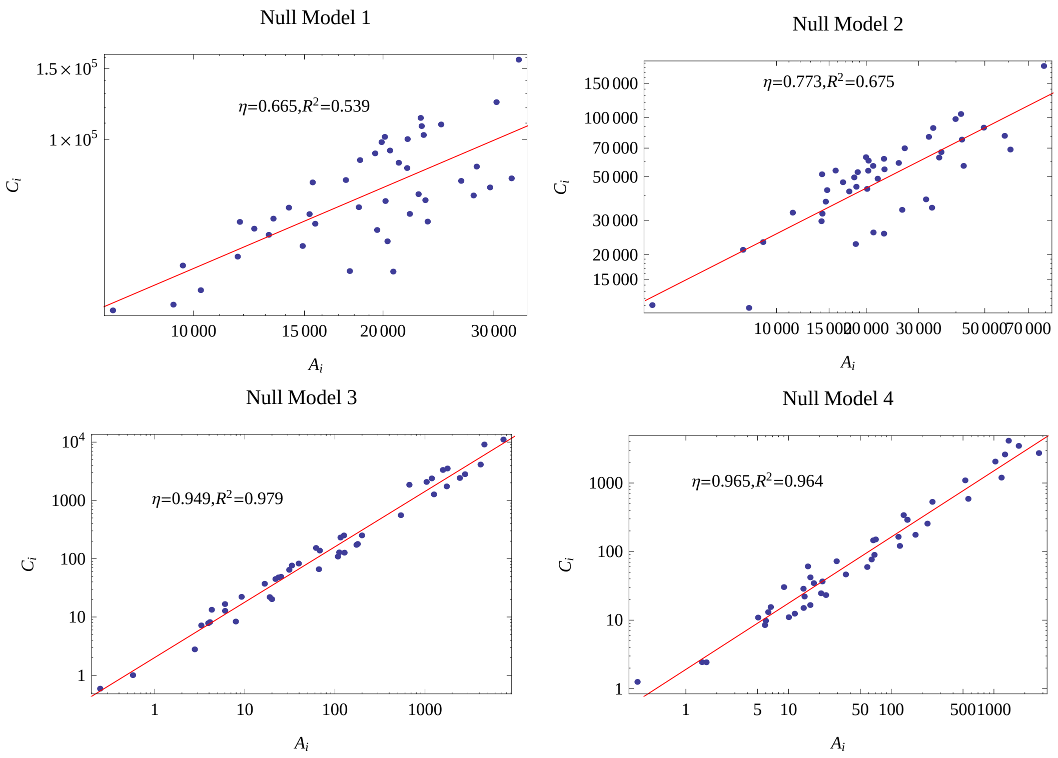

Figure S3. Allometric Scaling Patterns for Null Models based on the Mondego Network

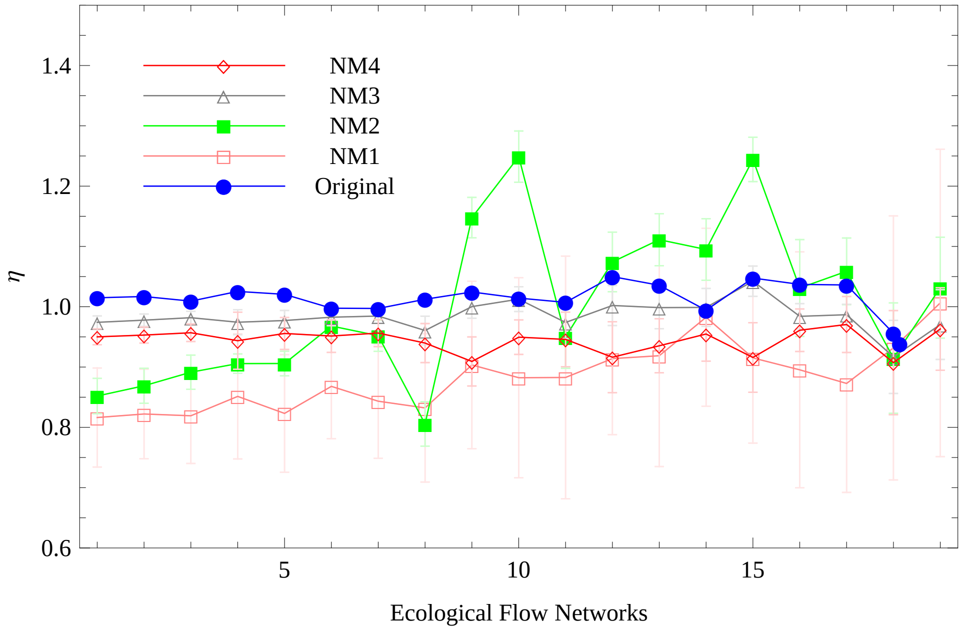

Figure S4. Comparison of Allometric Scaling Exponents of Original Networks with the Null Models All the empirical flow networks are sorted in the order of Table 1 in the main text along the horizontal axis(the left most network has largest number of nodes). The data points and error bars in all null models stand for the average values and standard deviations of exponents η respectively of fifty experiments.

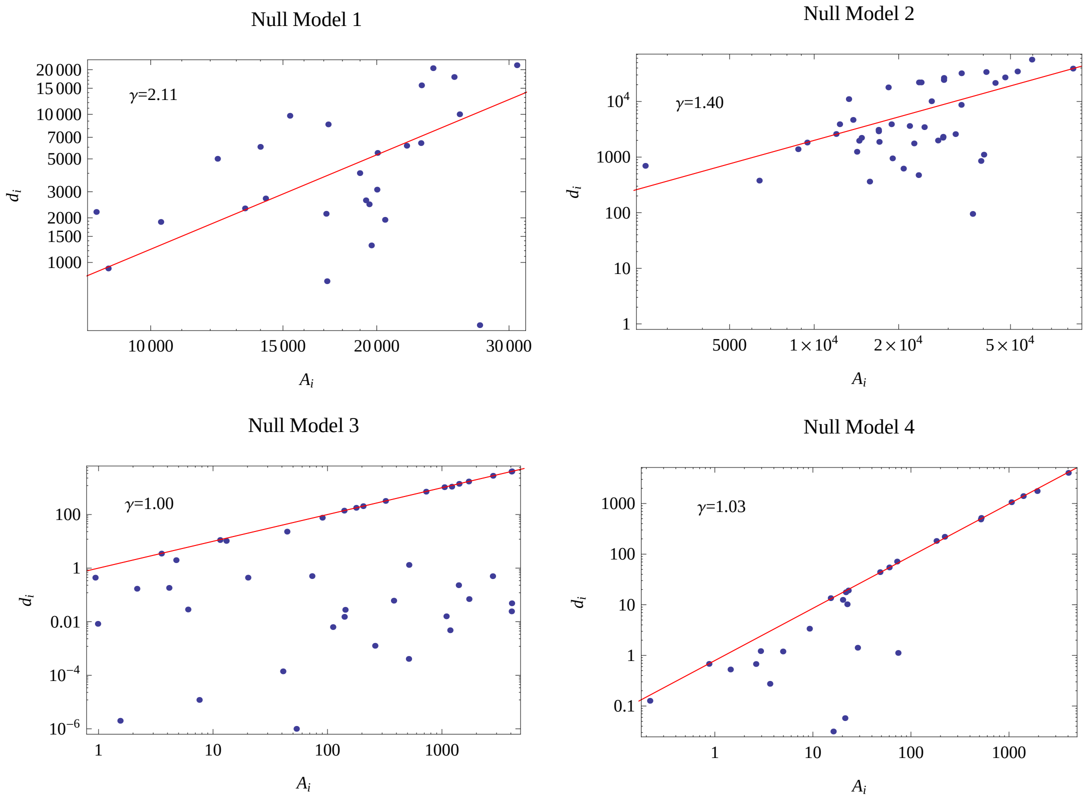

Figure S5. Dissipation Law for Null Models of Mondego Network

The dissipation laws are shown for the null model networks balanced by the method mentioned in Section A. The regression lines are obtained by neglecting the scatter points below the data cloud as outliers for Null Model 3 and Null Model 4.

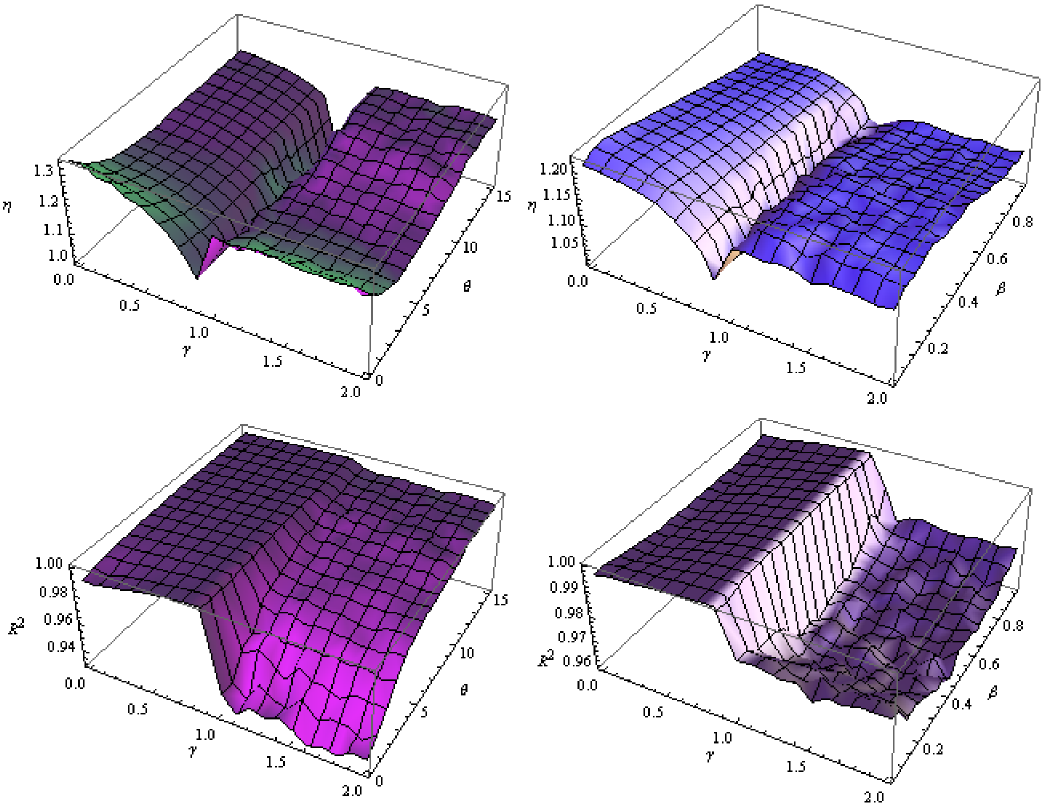

Figure S6. η and R^2 Change with θ,β and γ

For each combination of parameters, we generate 10 spanning trees to get the average value of η. The number of species, S, is set to 100 in all simulations

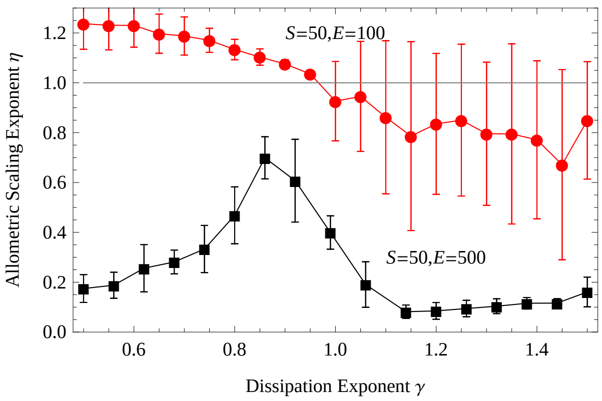

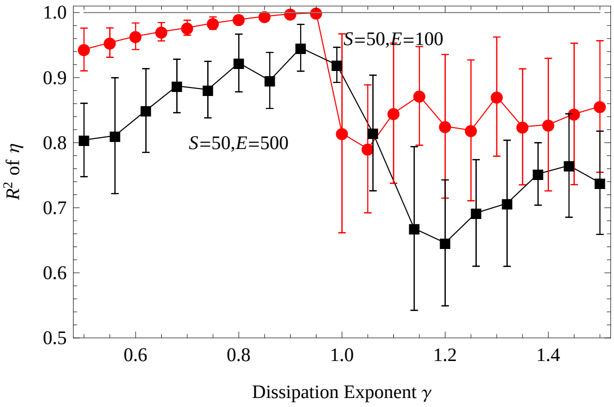

Figure S7. η and R^2 Change with γ for different random networks

All ηs are averaging for 10 random networks

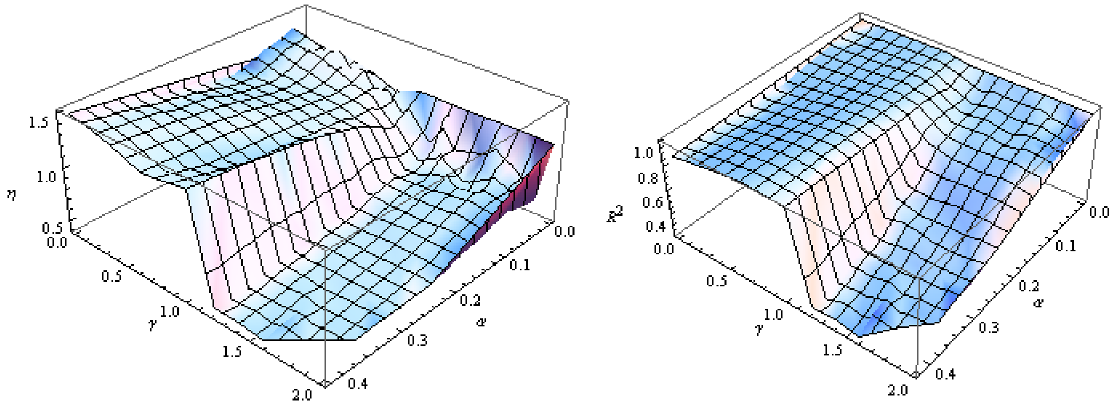

Figure S8. η and R^2 Change with γ and α on the Random Networks based on Spanning Trees All ηs are average results of 10 networks, the backbone trees are of the parameters $S = 100, \beta = 0.5, \theta = 5$.

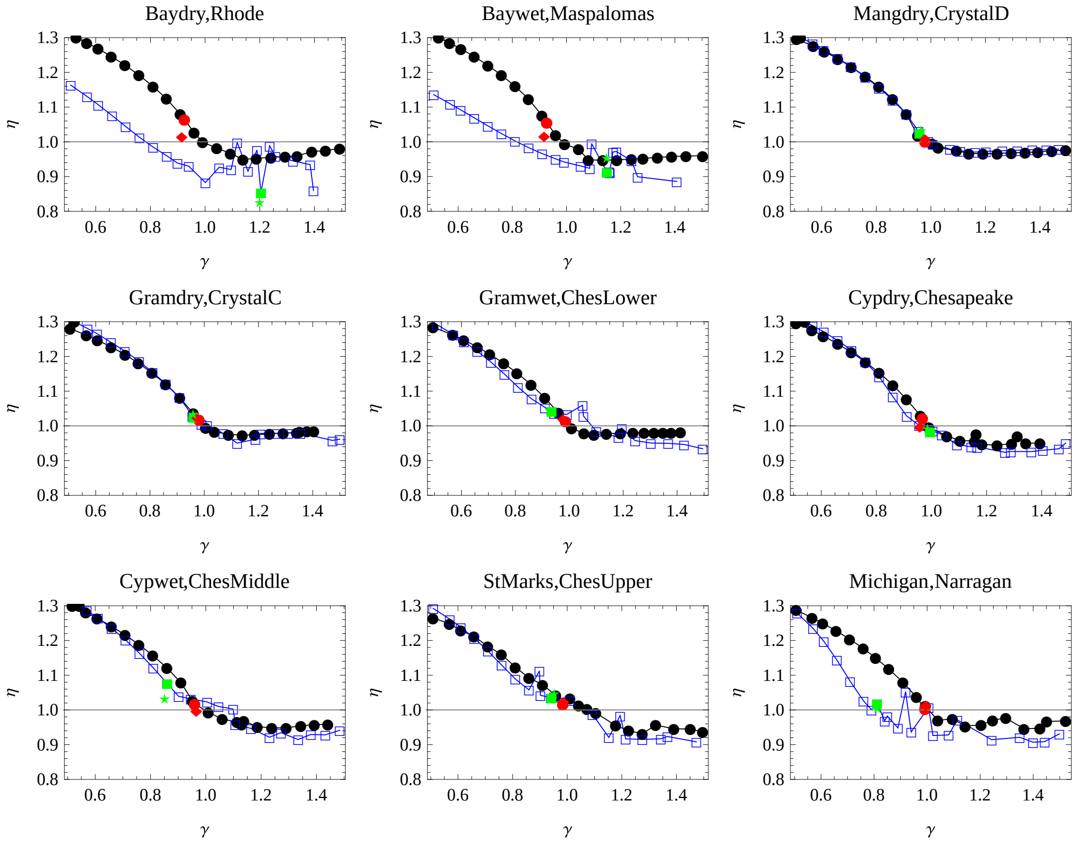

Figure S9. γ and η Relation Adjusted by the Flow Adjusting Algorithm on the Collected Ecological Networks

Each plot shows results of the Flow Adjusting Algorithm (FAA) on two empirical networks (black and blue curves), the red diamond and disk stand for the γ,η combinations for the original exponents and the adjusted results by the FAA on the original γ for the first ecological network; and the green star and square are for the second ecological network.

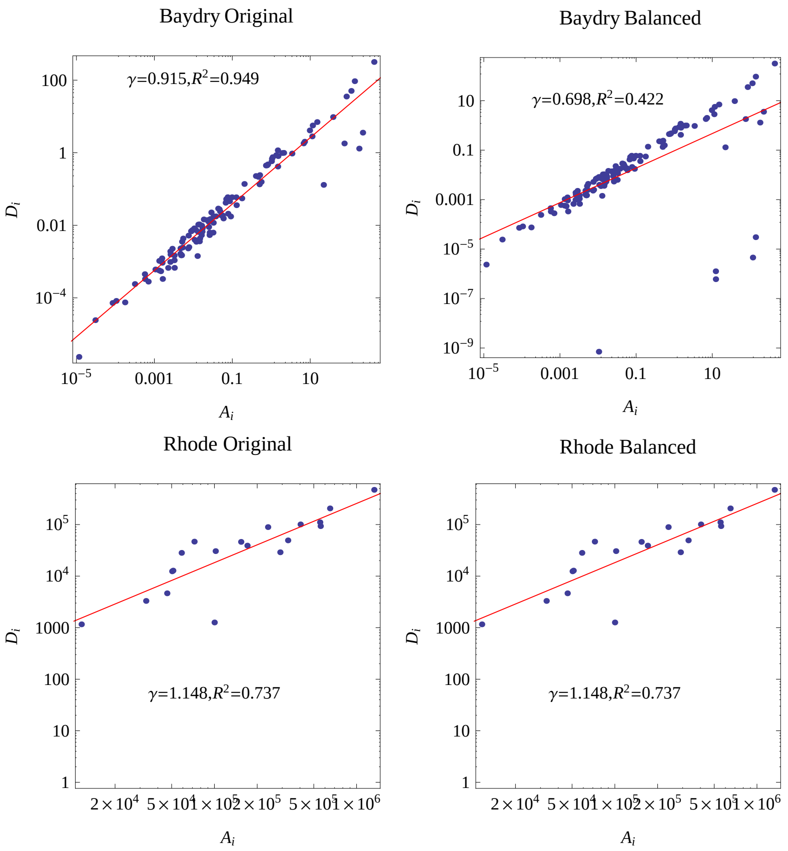

Figure S10. Dissipation Scaling Law for the Original and Balanced Flow Networks of Baydry and Rhode

List of Tables

S1 Unbalanced Flux for Each Ecological Network 19
S2 Comparison among Different Balancing Methods for Allometric Scaling Exponent 20

Table S1. Unbalanced Flux for Each Ecological Network

Network	Unbalanced Flux	Unbalanced Flux / Total Flux
Baydry	1.39601×10^{-4}	4.85587×10^{-8}
Baywet	3.89816×10^{-4}	9.28596×10^{-8}
Florida	3.89816×10^{-4}	9.28596×10^{-8}
Mangdry	4.73228×10^{-4}	7.47012×10^{-8}
Everglades	1.2759×10^{-3}	4.86597×10^{-8}
Gramdry	4.05957×10^{-4}	2.80647×10^{-8}
Gramwet	1.2759×10^{-3}	4.86597×10^{-8}
Cypdry	2.99341	7.50107×10^{-4}
Cypwet	2.29312	4.23941×10^{-4}
Mondego	1.263×10^{-3}	8.4865×10^{-8}
StMarks	1.039×10^{-4}	3.91837×10^{-8}
Michigan	4.89594×10^{-3}	1.0152×10^{-7}
Narragan	2.20391×10^{2}	4.15428×10^{-5}
ChesUpper	6.64	6.47632×10^{-6}
ChesMiddle	2.4079	1.0662×10^{-6}
Chesapeake	1.84615	3.68858×10^{-7}
ChesLower	1.078	6.11651×10^{-7}
CrystalC	6.7677×10^{2}	2.41561×10^{-2}
CrystalD	3.905×10^{1}	1.66493×10^{-3}
Maspalomas	0.	0.
Rhode	7.0477×10^{4}	1.33798×10^{-1}

Table S2. Comparison among Different Balancing Methods for Allometric Scaling Exponent

Network	Naive	Input	Output	Average
Baydry	1.010	1.010	1.010	1.020
Baywet	1.020	1.020	1.020	1.020
Florida	1.020	1.020	1.020	1.020
Mangdry	1.010	1.010	1.010	1.010
Everglades	1.020	1.020	1.020	1.020
Gramdry	1.030	1.030	1.030	1.030
Gramwet	1.020	1.020	1.020	1.020
Cypdry	0.998	0.996	0.996	0.996
Cypwet	0.997	0.998	0.997	0.997
Mondego	1.010	1.010	1.010	1.010
StMarks	1.020	1.030	1.030	1.030
Michigan	1.010	1.010	1.010	1.010
Narragan	1.010	1.010	1.010	1.010
ChesUpper	1.050	1.050	1.040	1.050
ChesMiddle	1.040	1.040	1.040	1.040
Chesapeake	0.994	0.994	0.994	0.994
ChesLower	1.050	1.050	1.040	1.050
CrystalC	1.040	1.030	1.050	1.040
CrystalD	1.040	1.040	1.040	1.040
Maspalomas	0.956	0.956	0.956	0.956
Rhode	0.828	0.717	0.750	0.699

"Input", "Output", and "Average" are short abbreviations for different methods mentioned in [2]
